# Isolated pineal region metastasis from lung adenocarcinoma with obstructive hydrocephalus: a case report

**DOI:** 10.1186/1752-1947-7-71

**Published:** 2013-03-14

**Authors:** Kenji Nemoto, Kazutetsu Aoshiba, Masayuki Itoh, Seitaro Semba, Takao Tsuji, Hideki Adachi, Hiroyuki Nakamura

**Affiliations:** 1 Department of Respiratory Medicine, Tokyo Medical University Ibaraki Medical Center, 3-20-1 Chuou, Ami, Inasiki, Ibaraki, 300-0395, Japan

**Keywords:** Chemotherapy, Hydrocephalus, Lung adenocarcinoma, Neuroendoscopic surgery, Pineal region metastasis, Stereotactic radiotherapy

## Abstract

**Introduction:**

Although the brain is a common site of metastasis from lung cancer, pineal region metastasis from lung adenocarcinoma is rare. Most cases of pineal metastases are asymptomatic, and are diagnosed by autopsy. Therefore, the management of pineal region tumors remains controversial. Here, we present a rare case of lung carcinoma presenting with pineal region metastasis and obstructive hydrocephalus as the first manifestation of the lung adenocarcinoma.

**Case presentation:**

A 63-year-old Japanese woman was referred to our hospital for treatment of a tumor of the pineal region associated with hydrocephalus. On admission, she was found to have a mass in her right lung on a chest radiograph. During the preoperative investigation, the patient began to show a progressively worsening level of altered consciousness. Therefore, neuroendoscopic surgery was performed as an emergency procedure, which resulted in improvement of the hydrocephalus and diagnosis of adenocarcinoma. A systematic investigation revealed adenocarcinoma of her right lung as the primary lesion. She was treated by a platinum-based chemotherapy regime. Stereotactic radiation to the pineal region was undertaken concurrently. After completion of the chemotherapy, the primary lesion and pineal region metastasis showed good partial response.

**Conclusion:**

The prognosis of pineal region metastasis is extremely poor, and only three patients with metastatic pineal region metastasis from lung cancer who were treated by chemotherapy have been reported. We performed neuroendoscopic surgery to obtain resolution of the obstructive hydrocephalus and the definite histological diagnosis. This resulted in improvement of the general condition of the patient, and the patient could be treated by chemotherapy and radiotherapy. We strongly believe that neuroendoscopic surgery was a good option in this case. This case report suggests that in the presence of an isolated pineal region tumor, metastasis should be considered a possible diagnosis, and careful examination for systemic malignant disease will be needed.

## Introduction

Although the brain is one of the most common sites of metastasis from lung cancer, metastasis to the pineal region is rather rare. In fact, metastasis to the pineal region has been estimated to account for only 0.4% of all intracranial metastatic tumors from lung adenocarcinoma in Japan [1]. A review of the literature revealed that the most common site of primary origin of a pineal metastasis is the lung and, histologically, small cell lung carcinoma is the most frequent type of cancer [2,3]. Our patient had adenocarcinoma; the association of this histological type with pineal metastasis seems to be rare. Most cases of pineal metastases are asymptomatic, and are diagnosed during autopsy [2,3]. Therefore, the clinical characteristics of, and therapeutic approach for, metastatic pineal region tumors have not been well discussed in the literature.

Here, we present a rare case of lung carcinoma presenting with pineal region metastasis and obstructive hydrocephalus as the first manifestation of the lung adenocarcinoma. In this case report, we discuss the clinical features of this unusual lesion from the viewpoint of differential diagnosis and therapeutic approach.

## Case presentation

**Figure 1:**
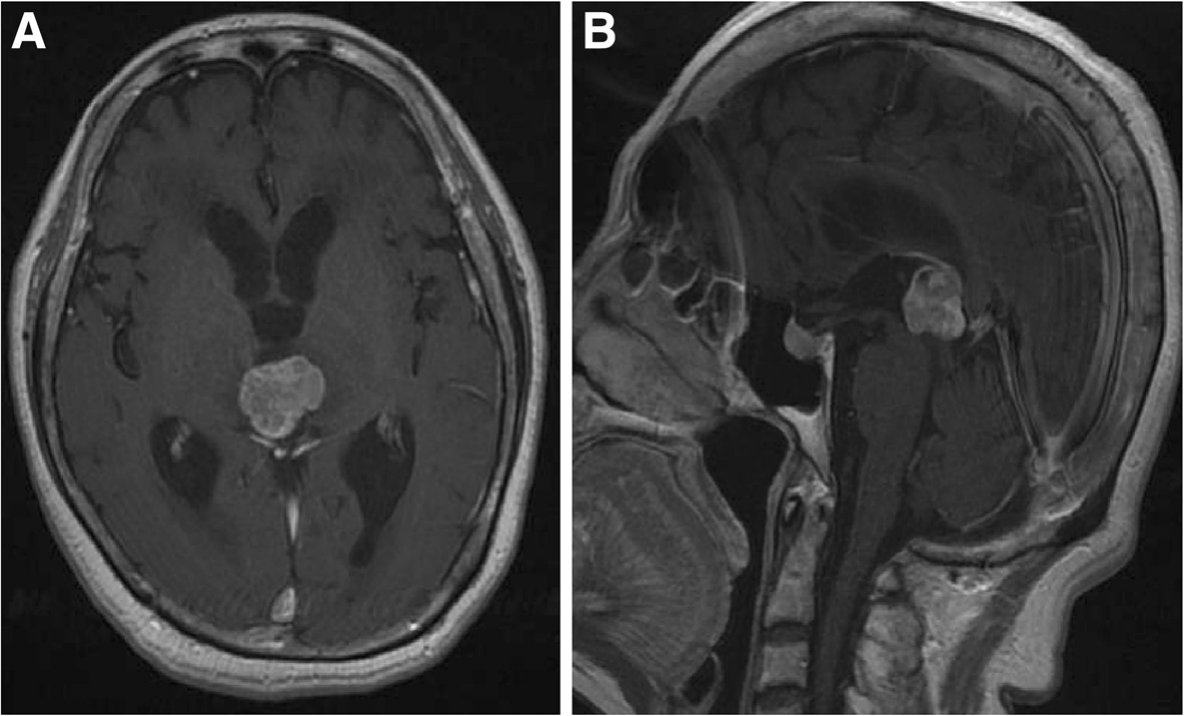
**Magnetic resonance imaging scan of the brain at diagnosis.** Magnetic resonance imaging with gadolinium injection showing a 25-mm pineal region tumor with heterogeneous enhancement associated with hydrocephalus on the T1-weighted axial image (**A**), and sagittal image (**B**).

**Figure 2:**
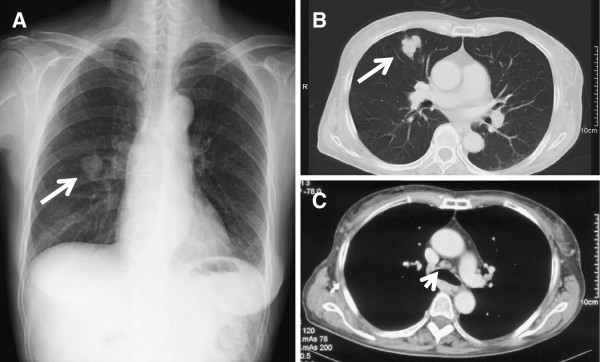
**Chest radiograph and computed tomography scan at diagnosis.** (**A**) Chest radiograph showing a mass shadow in the right middle zone (arrow). (**B**, **C**) Selected sections of a conventional computed tomography scan of the chest showing a 30-mm solitary mass in S^5^ of the right lung (arrow), and mediastinal lymphadenopathy (arrowhead).

**Figure 3:**
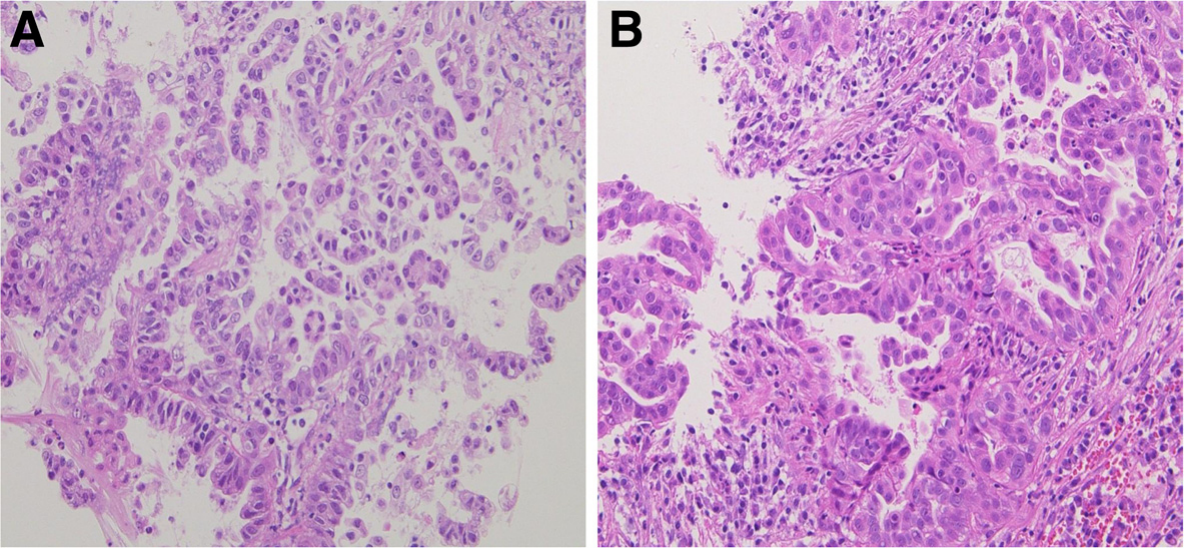
**Histology of the biopsy samples.** (**A**) Neuroendoscopic biopsy specimen of the tumor in the pineal region revealing adenocarcinoma with a papillotubular pattern (hematoxylin and eosin, ×200). (**B**) Transbronchial biopsy specimen in the lung tumor revealing adenocarcinoma similar to the pineal region tumor (hematoxylin and eosin, ×200).

A 63-year-old Japanese woman who had been in good health and had never smoked was admitted to a local hospital with 1-month’s history of gait disturbance, dementia, and urinary incontinence. She was referred to our hospital for suspected pineal region tumor with hydrocephalus. On admission to our hospital, neurological examination of the patient revealed no abnormalities, including evidence of visual disturbance; in addition, there were no features of hypopituitarism, such as secondary central diabetes insipidus or hypothyroidism. The standard blood workup also revealed no abnormalities. A computed tomography (CT) scan of her brain revealed hydrocephalus and a hyperdense space-occupying lesion in the pineal region. Magnetic resonance imaging (MRI) of her brain revealed a tumor in the pineal region measuring 25mm in diameter; the tumor was visualized as a hypointensity on T1-weighted images, as a uniform hyperintensity on T2-weighted images, and showed heterogeneous enhancement after gadolinium administration (Figure 1). There were no other intracranial space-occupying lesions. A chest radiography and CT showed a 30-mm nodule in the right middle lobe and ipsilateral mediastinal lymphadenopathy (Figure 2). The serum carcinoembryonic antigen (CEA) level was elevated to 247.9ng/mL (normal range <5.0ng/mL), whereas the serum levels of other tumor markers such as lactate dehydrogenase (LDH) and human chorionic gonadotropin (HCG) were within normal limits. During the preoperative investigation, the patient began to show a progressively worsening level of altered consciousness. Therefore, a neuroendoscopic third ventriculostomy with tumor biopsy was performed under general anesthesia as an emergency procedure. Histological examination of the tumor specimen revealed adenocarcinoma (Figure 3A). Subsequently, flexible fiberoptic bronchoscopy was performed, and a diagnosis of lung adenocarcinoma was established (Figure 3B). Based on the consistency of the histological characteristics between the lung tumor cells and pineal region tumor cells, the diagnosis of pineal metastasis from adenocarcinoma of the lung was established. Because an ^18^F-fluorodeoxyglucose positron emission tomography-CT revealed no other metastases, the lung adenocarcinoma was clinically characterized as T2aN2M1b, stage IV, according to the TNM classification of the International Union Against Cancer. The samples obtained from the brain and lung tumor cells revealed no mutation of the epidermal growth factor receptor gene.

**Figure 4:**
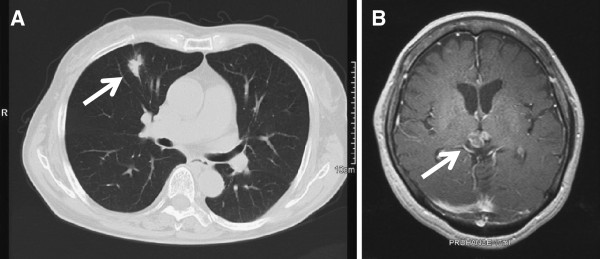
**Chest computed tomography scan and magnetic resonance imaging scan of the brain after the treatment.** (**A**) Chest computed tomography scan after the chemotherapy revealed shrinkage of the primary lesion (arrow), which was evaluated as a partial response. (**B**) An axial T1-weighted magnetic resonance imaging with gadolinium injection after the stereotactic radiation and chemotherapy showing resolution of hydrocephalus, and shrinkage of the pineal region tumor (arrow).

Postoperatively, with gradual improvement of the hydrocephalus, the patient was able to communicate and walk with assistance. Her performance status (Eastern Cooperative Oncology Group) was one, therefore, she was treated by chemotherapy using a combination of carboplatin (area under the concentration-time curve 5, day 1) plus pemetrexed (500mg/m^2^, Day 1) administered in 3-week cycles. Stereotactic radiation (45Gy in 15 fractions of 3Gy once daily) to the pineal region was undertaken concurrently. Grade 3 hematological side effects were observed, however, the patient tolerated the treatment. After six courses, she was assessed as showing good partial response by follow-up chest CT (Figure 4A) and brain MRI (Figure 4B). Currently, 12 weeks after completion of the chemotherapy, she remains alive, with no evidence of disease progression.

## Discussion

Primary lung cancer frequently metastasizes to various organs such as the lung, liver, bone and brain. Although the brain is a common site of metastasis from lung cancer, it is rare to encounter patients presenting with symptoms attributable to an isolated metastatic pineal region tumor even before the diagnosis of the primary lung cancer. In a large survey of 10,489 patients with intracranial metastatic tumors in Japan, metastases to the pineal region were found in only 37 patients (0.4%) [1], with cases showing a solitary mass detectable by CT and/or MRI, being even less frequent. According to a previous review, the most common site of the primary tumor is lung cancer, followed in frequency by breast cancer and malignant melanoma [2]. The most frequently reported histological type of lung cancer metastasizing to the pineal region is small cell carcinoma [2,3], although other histological types, including squamous cell carcinoma [4] and adenocarcinoma [5,6] have also been reported. In this case report the patient had adenocarcinoma; the metastasis of this histological type to the pineal region seems to be rare. The mechanism underlying the development of pineal metastasis is partially understood. Ortega *et al*. suggested hematogenous spread to the pineal body through the posterior choroidal arteries [7]. Because the pineal gland is excluded from the blood–brain barrier, it is more vulnerable to hematogenous metastasis from distant tumors [8].

A previous literature review actually identified a total of 63 such reported cases, and there were no other metastases within the brain in approximately half of these cases [3]. In the present case also, the pineal region was the only site of intracranial metastasis, which is a very interesting phenomenon. In this context, Kashiwagi *et al*. described, in relation to the mechanism of development of pineal metastasis, that one of the contributory factors was the special histological characteristics of the pineal body, namely, the numerous sinusoidal vessels with no perivascular glial sheets, which increases the vascular permeability [3]. The solitary metastasis to the pineal region also made it difficult to differentiate between metastatic intracranial disease and a primary tumor of the pineal region preoperatively. However, most patients with primary pineal tumors are reported to be younger than 30-years old [9], and metastasis should be considered in older patients [10]. In patients with a known history of malignancy, approximately 90% of all supratentorial lesions represent metastases [11]. This patient was 63-years old and had a primary lung tumor with elevated serum CEA levels, although it was not diagnosed preoperatively, both of which are consistent with the diagnosis of metastasis to the pineal region. In addition, our present patient did not show any elevation of the serum LDH or HCG levels, which enabled us to rule out germ cell tumor, the most frequently occurring primary tumor of the pineal gland [9].

We obtained histological specimens from both the tumor in the pineal region and the lung tumor by neuroendoscopic surgery and flexible fiberoptic bronchoscopy. The histological characteristics of the metastatic pineal region tumor were consistent with those of the lung adenocarcinoma. However, it may only be possible to obtain small fragments from pineal region tumors, as in this case, and in such a situation it may be difficult to differentiate between metastatic adenocarcinoma and germ cell tumors with malignant transformation into enteric-type adenocarcinoma. It is well known that a transformed germ cell tumor may express α-fetoprotein [12]. In the present case, no germ cell tumor elements or α-fetoprotein-positive cells were found in the mass.

According to previously reported cases, the prognosis of pineal region metastasis is extremely poor [13], and most such metastases are diagnosed by autopsy [2,3]. In fact, only three patients with metastatic pineal region metastasis from lung cancer who were treated by chemotherapy have been reported [3,14,15]. Therefore, it is important to obtain improvement of the symptoms caused by pineal lesion tumors and confirm the histological diagnosis as soon as possible. Surgical intervention, including ventricular drainage and ventriculoperitoneal shunting, is inherently associated with the risk of infection and peritoneal neoplastic dissemination; however, it is useful for alleviating symptoms due to hydrocephalus. In this case, we selected neuroendoscopic third ventriculostomy to obtain resolution of the obstructive hydrocephalus. This resulted in improvement of the general condition of the patient, and the patient could be treated by chemotherapy and radiotherapy. Hanada *et al*. reviewed the clinical data of 33 patients with metastatic pineal tumors diagnosed during life [13]; six of these patients underwent neuroendoscopic third ventriculostomy for hydrocephalus, with good control obtained in all the six patients. Furthermore, an additional advantage of the neuroendoscopic procedure is that tumor biopsy can be performed at the same time. Therefore, neuroendoscopic tumor biopsy rather than stereotactic biopsy or open surgery may be adequate for investigation of a pineal region tumor in patients of advanced age and poor medical condition. Therefore, we considered that neuroendoscopic surgery was a good option in this case.

## Conclusion

The pineal region is a rare intracranial site for metastasis, and metastasis to the pineal region without other brain metastases is even rarer. Our experience with this case suggests that in the presence of an isolated pineal region tumor, metastasis should be considered a possible diagnosis, and careful examination for systemic malignant disease will be needed. Although rare, metastatic pineal region tumors usually manifest as obstructive hydrocephalus, as in the present case. Selection of neuroendoscopic surgery may provide immediate relief from hydrocephalus and also a definitive histological diagnosis.

## Consent

Written informed consent was obtained from the patient for the publication of this case report and its accompanying images. A copy of the written consent is available for review by the Editor-in-Chief of this journal.

## Competing interests

The authors declare that they have no competing interests.

## Authors’ contributions

KN performed the literature search and drafted the manuscript. MI and KA conceived the case report and provided guidance for drafting the manuscript. SS, TT, HA, and HN participated in its design and co-ordination and helped to draft the manuscript. All authors read and approved the final manuscript.
